# New Astringent Preparation

**Published:** 1843-09

**Authors:** 


*New Astringent Preparation.*—By digesting rhatany in sulphuric ether a brown extract is obtainable, perfectly soluble in distilled water, and when placed on the tongue giving a sensation of great astringency, followed by heat and dryness. This extract, invented by a Mr. Tissier, of Lyons, has been employed with success in that city in passive hæmorrhages, particularly those consequent on non-contraction of the uterus, as after prolonged labours and miscarriages. It has also been used with advantage for leucorrhœa, blennorrhœa, gleet, &c. The dose in which it has hitherto been employed is a tablespoonful of a mixture composed of from five to ten grains of the extract in six ounces of some appropriate vehicle; in leucorrhœa, topical injections are recommended of from two to five grains of extract in a pint of barley-water. The presence of this preparation in the stomach gives rise, generally, to a sensation of heat in the epigastrium, though this rarely proceeds so far as to become painful; great thirst, and a pulse often as full as in gastritis, also prevail. These symptoms are, however, transient, and readily quelled by lemonade or other mild drinks. Should the injection irritate the urethra too greatly, it is only necessary to suspend its use for a short time.—*Gazette des Hôpitaux*, June 6th.—*Lond. Lancet.*

